# Transcriptomic analysis of cecal mucosal immunity in SPF White Leghorn chicks infected with precocious and parent strains of *Eimeria tenella*

**DOI:** 10.3389/fimmu.2026.1954459

**Published:** 2026-09-28

**Authors:** Wenyu Ma, Kexin Du, Tingting Yi, Xuefei Liang, Shuaijing Niu, Xuewei Liu, Jian An, Deqi Yin, Qiuming Li

**Affiliations:** Coccidia Laboratory, College of Animal Science and Technology/College of Veterinary Medicine, Beijing University of Agriculture, Beijing, China

**Keywords:** *Eimeria tenella*, parent strain, precocious line, mucosal immunity, transcriptome

## Abstract

**Introduction:**

*Eimeria tenella* (*E. tenella*) preferentially invades the cecum of young chickens and causes enormous economic losses to the global poultry industry.

**Methods:**

In this study, we established chicken infection models for the virulent parent strain and its precocious attenuated line, and collected samples at 2 dpi and 6 dpi as two critical time points. By integrating pathogenicity evaluation, transcriptome sequencing, detection of mucosal immune-related indicators, and homologous challenge protection assays, we systematically revealed the distinct molecular mechanisms underlying strain-specific differences in pathogenicity and immune regulation.

**Results:**

Pathogenicity results showed that increased infection dosages suppressed weight gain and aggravated bloody diarrhea and oocyst shedding in both strains. In particular, infection with 1 × 10^5^ sporulated oocysts of the parent strain caused massive chick mortality, while the precocious line exhibited markedly lower virulence. Transcriptomic data revealed that gametogony (6 dpi) represented the peak of host immune response. The parent strain persistently overactivated the NF- κB - mediated positive feedback cascade of coagulation and complement as well as ECM remodeling, triggering steroid metabolic disorder and antioxidant exhaustion, which ultimately induced severe hemorrhagic necrosis of the cecum. By contrast, the precocious line specifically activated the PPAR - γ signaling pathway to negatively restrain excessive inflammation, accompanied by enriched TLR signaling and leukocyte transendothelial migration pathways, thereby forming an immune cascade of "lipid anti - inflammation - pathogen elimination - mucosal repair". Immunoprotection trials verified that priming with 1 × 10^4^ sporulated oocysts of the precocious line significantly alleviated cecal lesions and reduced oocyst output upon secondary challenge, conferring stable mucosal immune protection. The expression trends of seven hub genes (*PPARG, PLIN1, CYP1A1, THBS1, FMO4, CYP2C18, CXCL14*) detected via qRT - PCR were consistent with RNA - seq data.

**Conclusions:**

This study preliminarily elucidated a dual regulatory paradigm consisting of NF - κB - mediated tissue injury and PPAR - γ - dependent anti - inflammatory responses, refined the mucosal immune theoretical framework for commercially available precocious attenuated strains, and provided candidate molecular targets for targeted anti - coccidial intervention in chickens.

## Introduction

Avian coccidiosis is an intestinal parasitic disease caused by protozoans of the genus *Eimeria* (1). Among all chicken coccidia, *Eimeria tenella* (*E. tenella*) exerts the highest pathogenicity in chicks aged 15 to 50 days (2). Coccidiosis incurs annual economic losses exceeding 14 billion US dollars worldwide, posing a severe threat to the sustainable development of the poultry industry (3). The parasite transmits mainly via the fecal - oral route, with lesions specifically localized in the cecum. Clinically infected chicks manifest reduced feed intake, intestinal inflammation, hemorrhagic diarrhea and growth retardation; severe infection may result in extensive cecal necrosis and even massive chicken death (4). Although live attenuated vaccines derived from precocious lines have been commercially produced, the molecular regulatory network of host mucosal immunity responsible for their low virulence and high immunogenicity remains poorly characterized.

High - throughput RNA - seq provides a powerful tool to dissect the molecular crosstalk between coccidian parasites and their avian hosts, and multi-omics research focusing on *E. tenella* has expanded rapidly in recent years (5, 6). Previous transcriptomic analyses indicated that overexpression of malate dehydrogenase is closely associated with parasite endogenous development, host cell invasion and drug resistance formation (7). Single - cell RNA sequencing demonstrated that *E. tenella* infection drives prominent epithelial apoptosis, accompanied by reduced abundance of cell subsets maintaining mitochondrial and cytoplasmic homeostasis (8). Widespread dynamic m^7^G mRNA methylation during oocyst sporulation was further uncovered. This epigenetic modification modulates the transcription of metabolic and developmental genes and directly determines oocyst infectivity (9). Existing comparative transcriptomics between precocious lines and parent strains solely focused on merozoite - derived virulence genes such as microneme, rhoptry and dense granule proteins (10). These parasite - centric investigations fail to explain the distinct phenotypic differences: mild intestinal damage and superior protective immunity induced by precocious line infection.

Current relevant research bears prominent limitations. Most omics studies only adopt a single infection time point, lacking temporal comparisons of host cecal mucosal immunity across schizogony and gametogony. Moreover, transcriptomic profiling of precocious lines largely concentrates on parasite genes while neglecting dynamic host immune remodeling along parasite developmental stages. Schizogony at 2 dpi and gametogony at 6 dpi constitute two critical time windows determining strain virulence and immunogenicity. To date, no research has simultaneously compared host transcriptional profiles induced by parent and precocious lines at these two key stages, creating a critical knowledge gap hindering in - depth interpretation of precise anti - coccidial strategies. In the present study, we systematically evaluated the *in vivo* pathogenic differences between parent and precocious *E. tenella* strains, employed RNA - seq to compare cecal transcriptional profiles at 2 dpi and 6 dpi post-infection, and elucidated the host molecular mechanisms driving divergent pathogenicity, offering theoretical evidence for deciphering the immunological basis of commercial precocious live vaccines and screening novel targeted anti - coccidial molecular markers.

## Materials and methods

### Ethical statement

All animal procedures in this experiment were reviewed and approved by the Institutional Animal Welfare and Ethics Committee of Beijing University of Agriculture (Ethics approval No. BUA2022080). All animal procedures were performed in accordance with the regulations and guidelines of the committee to ensure humane treatment of chicks.

### Animals

A total of 150 seven-day-old male SPF White Leghorn chicks with body weight variation ≤ ± 5 g were purchased from Beijing Boehringer Ingelheim Vital Animal Biotechnology Co., Ltd. After eliminating weak and underdeveloped individuals, the qualified chicks were randomly allocated to four parallel experimental batches for pathogenicity determination, transcriptome sequencing, sample collection and immune protection assay, respectively. All chicks were raised under consistent feeding conditions, including a constant temperature of 22 ± 2°C and a 12 h light/12 h dark photoperiod, and were continuously fed commercial coccidia-free and antibiotic-free complete chick feed (XTC07YJ-001, Jiangsu, China) throughout the entire experimental period.

### *Eimeria tenella* oocysts

The virulent parent Henan strain and its derived precocious line of *E. tenella* were preserved in the Chicken Coccidiosis Laboratory, College of Animal Science and Technology, Beijing University of Agriculture. Oocysts were propagated one month prior to experiments, sporulated in 2.5% potassium dichromate solution, and stored at 4°C for subsequent use. The prepatent period of the parent strain was 143 h, while repeated chicken passages shortened the prepatent period of the precocious line to 121 h.

### Experimental design

As shown in **Figure 1A**, in the first phase, the pathogenicity test was performed on the parental strain and precocious line of *E. tenella*. A total of 63 healthy 28-day-old chicks were randomly divided into seven groups, with nine chicks per group. The experimental groups consisted of one blank control group, and three challenge dose subgroups for the parental strain and precocious line of *E. tenella*, respectively, with inoculation doses of 1×10³, 1×10^4^ and 1×10^5^ sporulated oocysts per bird. At 28 days of age, chicks in all challenge groups were orally inoculated with the corresponding dose of sporulated oocysts, while chicks in the blank control group received an equal volume of sterile normal saline by oral gavage. The cecal tissues were collected from chicks in the blank control group and the two groups infected with 1×10^4^ oocysts of the two strains at 2 dpi and 6 dpi for pathological section preparation. At 7 dpi, body weight gain, mortality rate, bloody stool score and oocyst per gram of cecal contents (OPG) of chicks in each group were determined.

**Figure 1:**
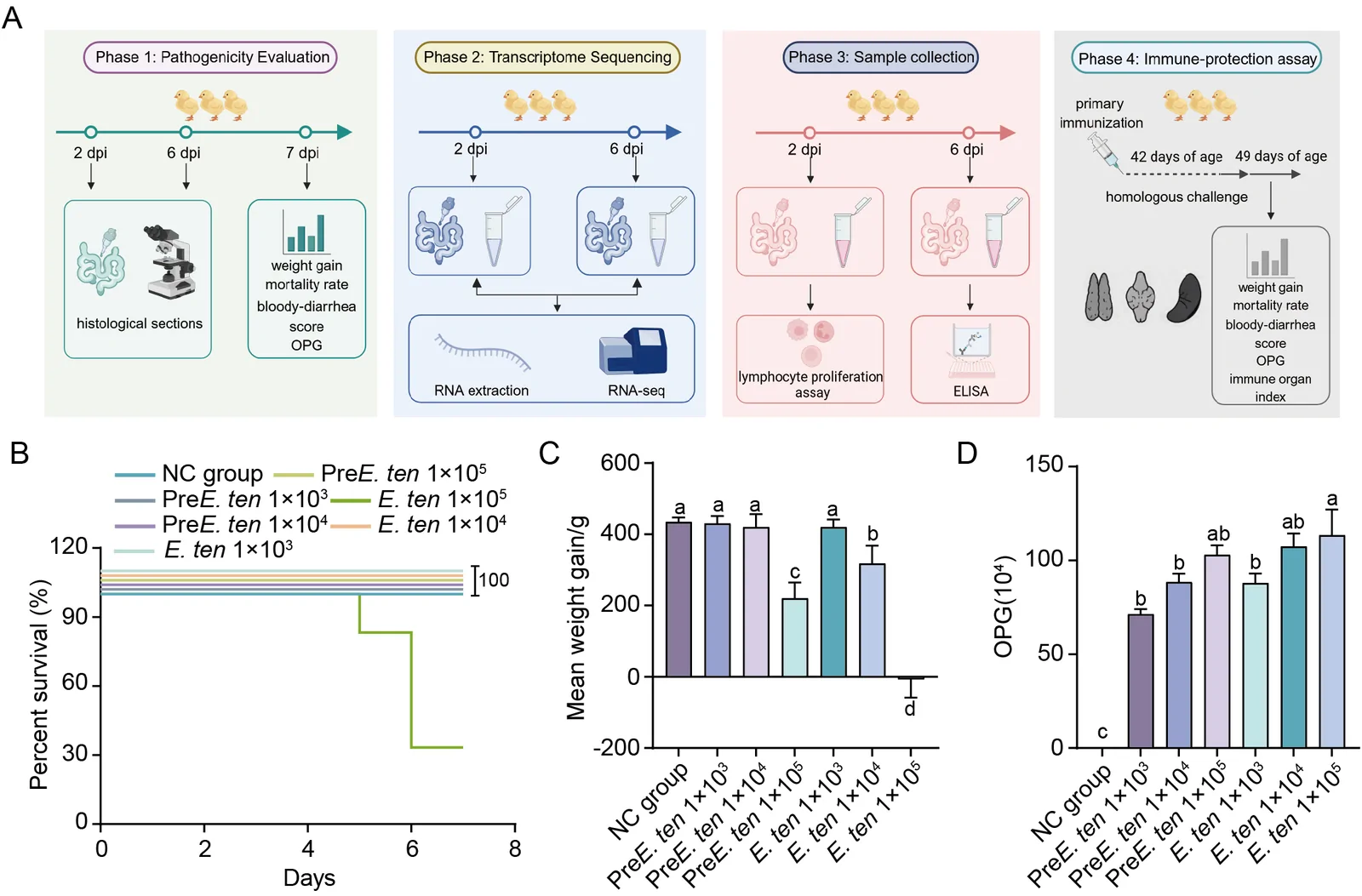
Pathogenicity detection of sporulated oocysts of *E. tenella* precocious line and parent strain. **(A)** The schematic of experimental design. **(B)** Effects of different dosages of precocious line and parent strain oocysts on the survival rate of chicks during pathogenicity detection. **(C)** Effects of different dosages of precocious line and parent strain oocysts on body weight gain of chicks during pathogenicity detection. **(D)** OPG of cecal contents in each group. Data are presented as mean ± SE (n = 9); Values marked with different lowercase superscript letters **(a-d)** within the same column indicate significant differences at *P* < 0.05. The figure is created with BioRender.com.

In the second experimental phase, a separate batch of 15 SPF chicks aged 28 days was used to generate samples for transcriptome sequencing. The chicks were randomly assigned to one blank control group (C group) and four infection groups: precocious line-infected groups sampled at 2 and 6 dpi (PreE2 and PreE6), and parent strain-infected groups sampled at 2 and 6 dpi (E2 and E6), with three chicks in each group. Chicks in all infection groups received an oral inoculation of 1 × 10^4^ sporulated oocysts of the corresponding strain, whereas chicks in the blank control group were given an equal volume of sterile normal saline. Segments of cecal tissue (2 cm in length) were harvested at 2 dpi and 6 dpi. The tissues were cut longitudinally, and cecal contents were thoroughly washed away using pre-cooled sterile PBS. The tissues were then treated with Trizol reagent (Sangon Biotech, Shanghai, China) and stored in liquid nitrogen for subsequent RNA extraction and transcriptome sequencing.

In the third phase, animal experiments were performed to collect cecal samples from chickens infected with the parental strain and precocious line of *E. tenella*. A total of 36 healthy 28-day-old chicks were randomly assigned into six groups (six chicks per group), including a negative control group, parental strain infection group(*E. ten*), and precocious line infection group(Pre*E. ten*). At 28 days of age, chicks in the infection groups were orally inoculated with 1 × 10^4^ sporulated oocysts of the corresponding parental strain or precocious line. Three chicks from each group were humanely euthanized at 2 and 6 dpi and cecal tissues were harvested for subsequent lymphocyte proliferation assay and ELISA detection.

In the fourth phase, the priming-immunization and homologous challenge protection assay was carried out using the parental strain and precocious line of *E. tenella*. Thirty-six healthy 28-day-old chicks were randomly divided into six groups with six chicks per group, including negative control group (NC), low-dose parental strain priming group, high-dose parental strain priming group, low-dose precocious line priming group, high-dose precocious line priming group, and positive challenge control group (PC). Primary immunization was implemented at 28 days of age. Chicks were orally vaccinated with 1 × 10³ or 1 × 10^4^ sporulated oocysts of parental strain or precocious line respectively. All primed groups received homologous challenge with 1 × 10^5^ sporulated oocysts of parental strain at 42 days of age. The PC group had no prior immunization and was only challenged with the same dose of parental strain oocysts at 42 days of age. Body weight gain, mortality rate, bloody-fecal score, OPG and immune organ indices were determined at 49 dpi.

### Bloody stool scoring

Bloody stool conditions of each experimental group were observed daily from day 4 to day 7 post-infection. A scoring scale ranging from 0 to 4 was adopted in accordance with the method described by Youn et al. (11). 0 denoted normal feces with no visible blood, while scores of 1, 2, 3 and 4 corresponded to 25%, 26–50%, 51–75% and >75% bloody content among total fecal output, respectively.

### Quantification of oocysts per gram of cecal contents

At 7 dpi, 2 g cecal content samples were collected from each group. Oocyst counting was performed using the McMaster technique as described by Saber et al. (12). 2 g of fresh feces was suspended and mixed in 58 mL of saturated sodium chloride solution, thoroughly mixed. The resulting suspension was transferred using a Pasteur pipette into a McMaster counting chamber and allowed to settle for 5 min before oocyst enumeration.

### Measurement of immune organ indices

Chicks were weighed before euthanasia. The intact spleen, bursa of Fabricius and thymus were aseptically dissected, with excess fat and connective tissues removed using forceps and scissors. Fresh organ weights were recorded to calculate immune organ indices, which were defined as organ weight/live body weight.

### RNA extraction and transcriptome sequencing

Total RNA was extracted using the Total RNA Extractor kit (Sangon Biotech, Shanghai, China) following the manufacturer’s protocol, and treated with RNase-free DNase I to eliminate genomic DNA contamination. RNA integrity was assessed via 1.0 % agarose-gel electrophoresis. RNA purity and concentration were further determined using a NanoPhotometer^®^ spectrophotometer (IMPLEN, CA, USA) and a Qubit^®^ 2.0 Fluorometer (Invitrogen, USA). All samples showed OD_260_/_280_ ratios between 1.95 and 2.10, confirming high-quality input material for transcriptome sequencing. Qualified RNA samples were subsequently sent to Sangon Biotech (Shanghai) Co., Ltd. for library preparation and paired-end sequencing.

A total amount of 1 μg RNA per sample was used as input material for the RNA sample preparations. Sequencing libraries were generated using VAHTSTM mRNA-seq V2 Library Prep Kit for Illumina^®^ following manufacturer’s recommendations. MRNA was enriched from total RNA using poly-T oligo magnetic beads and fragmented. First-and second-strand cDNA were successively synthesized, followed by end-repair, 3′-end A-tailing and adaptor ligation. Target cDNA fragments (150~200 bp) were purified with AMPure XP beads, then treated with USER enzyme before PCR amplification using Phusion high-fidelity DNA polymerase. PCR products were purified by AMPure XP system, and library quality was verified on the Agilent Bioanalyzer 2100 system.

Qualified libraries were quantified, pooled and subjected to paired-end sequencing on the Illumina NovaSeq platform. FastQC (v0.11.2) was applied for raw-read quality assessment. Trimmomatic (v0.36) was used to filter raw reads. Sequencing quality control thresholds were set as Q20 ≥ 95% and Q30 ≥ 85%. Clean reads were separately aligned to two independent reference genomes using HISAT2 (v2.0) with default parameters. Specifically, reads were mapped to the chicken reference genome (Gallus gallus, GCF_016700215.2_GRCg7w) and the *E. tenella* reference genome (GCF_000499545.2_ETH001) in two independent alignment procedures. RSeQC (v2.6.1) was adopted to summarize alignment statistics. Qualimap (v2.2.1) was used to evaluate read-distribution homogeneity and genome-structure alignment. BEDTools (v2.26.0) was applied for statistical analysis of gene-coverage ratio. ANOSIM was performed based on the Bray-Curtis distance matrix to evaluate the dissimilarity of gene expression profiles among different groups. DESeq2 (v1.12.4) was used to identify differentially expressed genes (DEGs). DEGs were screened with the criteria of ∣log_2_FC∣ > 2 and *P-values* < 0.05, followed by functional enrichment analyses of Gene Ontology (GO), Kyoto Encyclopedia of Genes and Genomes (KEGG) and Eukaryotic Orthologous Groups (KOG) to identify significantly enriched GO terms, signaling pathways and functional categories. Statistical significance was defined as *P-values* < 0.05.

### Validation of RNA-seq data via quantitative real-time PCR

Total RNA isolated from caecal tissues was reverse-transcribed using the HiFiScript All-in-one RT Master Mix for qPCR kit (CWBIO, Jiangsu, China), and the resulting cDNA was used as the template for RT-qPCR. RT-qPCR was performed on a real-time PCR instrument (Agilent, La Jolla, CA, USA) following the manufacturer’s instructions for SuperStar Universal SYBR Master Mix(CWBIO, Jiangsu, China), to validate the mRNA expression levels of seven candidate genes (*PPARG, PLIN1, CYP1A1, THBS1, FMO4, CYP2C18, CXCL14*) identified from transcriptome data, with β-actin serving as the reference gene. The amplification program was set as follows: initial denaturation at 95°C for 30 s, followed by 40 cycles of 95°C for 15 s and 60°C for 30 s, with a melting curve generated from 65°C to 95°C. The relative gene expression was calculated using the 2^-ΔΔCt^ method. Primer sequences are listed in **Table 1**. All qRT-PCR reactions were conducted in three technical replicates.

**Table 1:** Primer sequences for qRT-PCR.

Gene name	Forward primer (5′→3′)	Reverse primer (5′→3′)
PPARG	TGACAGCGCCAGAGATTACA	AAATAGGGAGGAGAAGGAGGCT
PLIN1	ATGGAAGGGCCAAGGAGAAC	GACCTCTTTGGTGCTGGTGT
CYP1A1	GCGAACCACGACGAGAAGA	GTACTTCATGGTCAGCCCGT
THBS1	CAGCTTTACGTGGGGTGTGA	ACATTCTGCAACAGTCCCTGG
FMO4	TTCTGGCTGCATTTTGCGTG	CGCGGTCTGCTTTGTCTGTA
CYP2C18	GAGGACACATGCCTATCGGG	GGGTCAAAGGGCAGTCTCTT
CXCL14	CTTAGCCAGTGCAGAAGGAGT	CACTGTGTTCTGGCGTTTGG
β-actin	TGATATTGCTGCGCTCGTTG	ATACCAACCATCACACCCTGA

### Histopathological analysis

Cecal tissues (5 cm long) were collected at 2 and 6 dpi, fixed in 4% tissue and cell fixative solution (Cat. No.: PQ0662, Solarbio, Beijing, China) for 72 h, processed into paraffin sections and stained with hematoxylin and eosin for microscopic observation. Remaining cecal tissues were preserved for subsequent immune functional assays.

### Isolation and proliferation assay of cecal lymphocytes

Cecal tissues were harvested under aseptic conditions, minced, homogenized, and subjected to density gradient centrifugation to isolate lymphocytes. After washing, cells were resuspended for subsequent experiments. A total of 1 × 10^5^ lymphocytes suspended in 200 μL medium were seeded into each well of a 96 - well plate, followed by stimulation with ConA (Biosharp, Beijing, China) for 72 h. Optical density at 490 nm was detected according to the manufacturer’s instructions of the MTS kit(Promega, Beijing, China), and the stimulation index (SI) was calculated accordingly.

### Enzyme-linked immunosorbent assay

Cecal tissues were homogenized in nine volumes of pre - cooled PBS, and homogenates were centrifuged at 5000 rpm for 15 min at 4 °C to collect supernatants. The concentrations of secretory immunoglobulin A (SIgA) in cecal contents were determined using a chicken SIgA ELISA kit (mlbio, Shanghai, China), with absorbance measured at 450 nm.

### Statistical analysis

All experiments were performed in three independent biological replicates. Statistical analyses were carried out using GraphPad Prism 10.0 and SPSS 17.0 software. All data were presented as mean ± standard error (SE). Two - tailed unpaired Student’s t - test was used for comparisons between two groups, while one-way analysis of variance (ANOVA) was applied for multiple group comparisons. Values within the same column marked with different superscript lowercase letters (a - d) indicate significant differences at *P* < 0.05. Statistical significance levels were set as **P < 0.01 and ***P < 0.001.

## Results

### Pathogenicity detection

Significant pathogenic differences were observed between the parent strain and precocious line of *E. tenella*. As shown in **Figure 1B**, mortality occurred in chicks infected with 1 × 10^5^ sporulated oocysts of the parent strain, with a final survival rate of 33.3%, whereas no deaths were recorded in all other experimental groups. The body weight changes of chicks are presented in **Figure 1C**. Compared with the NC group, the 1 × 10^5^ PreE. ten group, 1 × 10^4^ E. ten group and 1 × 10^5^ E. ten group exhibited significantly reduced body weight gain. Notably, infection with 1 × 10^5^ parent strain oocysts induced negative body weight growth in chicks. OPG quantification of fecal samples revealed that both parent and precocious line infection groups showed statistically higher OPG values than the NC group, and the OPG levels increased in a dose-dependent manner (**Figure 1D**). Bloody stool scoring results demonstrated that chicks in the 1 × 10^4^ and 1 × 10^5^ dosage groups of both strains developed bloody stool starting at 4 dpi, and the total bloody stool scores increased with the elevated infection dosage (**Table 2**).

**Table 2:** Statistical analysis of bloody stool scores after primary coccidial infection in different groups.

Group	Oocyst infection dose	84 h	93 h	108 h	117 h	132 h	141 h	156 h	165 h	Score of bloody feces
NC	0	0	0	0	0	0	0	0	0	0^c^
Pre*E. ten*	1.0×10^3^	0	0	0	0	0	0	0	0	0^c^
Pre*E. ten*	1.0×10^4^	2	3	1	0	0	0	0	0	0.75 ± 1.1^bc^
Pre*E. ten*	1.0×10^5^	3	4	2	1	0	0	0	0	1.25 ± 1.5^ab^
E. ten	1.0×10^3^	0	0	0	0	0	0	0	0	0^c^
E. ten	1.0×10^4^	0	1	3	2	1	0	0	0	0.87 ± 1.1^bc^
E. ten	1.0×10^5^	0	2	4	4	3	2	1	1	2.13 ± 1.4^a^

Data are presented as mean ± SE (n = 9); Values marked with different lowercase superscript letters (a-d) within the same column indicate significant differences at *P*  < 0.05.

### Transcriptomic data analysis

A total of 15 cecal transcriptome libraries were constructed and sequenced. The results yielded 36,376,946 to 58,106,532 paired-end raw reads (150 bp) per library, with GC contents ranging from 46.44% to 48.89%. Low-quality reads, ambiguous bases, and adapter sequences were filtered using Trimmomatic software, generating 36,332,048 to 58,033,254 valid clean reads with a total data volume of 97.9 Gb. The Q20 and Q30 values of all clean data ranged from 97.36% to 97.91% and 93.54% to 94.65%, respectively (**Supplementary Files 1**, **2**). These quality control indicators confirmed that the transcriptomic data were reliable and qualified for subsequent analysis of cecal mucosal immune responses.

ANOSIM showed that the R value was close to 1, indicating significantly higher intergroup differences than intragroup differences (**Figure 2A**). PCA further revealed distinct transcriptional profiles among the C, PreE6, and E6 groups (**Figure 2B**). Venn diagram analysis identified 18,281 conserved genes across all five groups. Specifically, the parent strain-infected groups (E2, E6) possessed 410 and 352 unique expressed genes, while the precocious line-infected groups (PreE2, PreE6) had 312 and 501 unique expressed genes, respectively, providing fundamental data for elucidating the differential cecal mucosal immune responses induced by the two virulent strains (**Figure 2C**).

**Figure 2:**
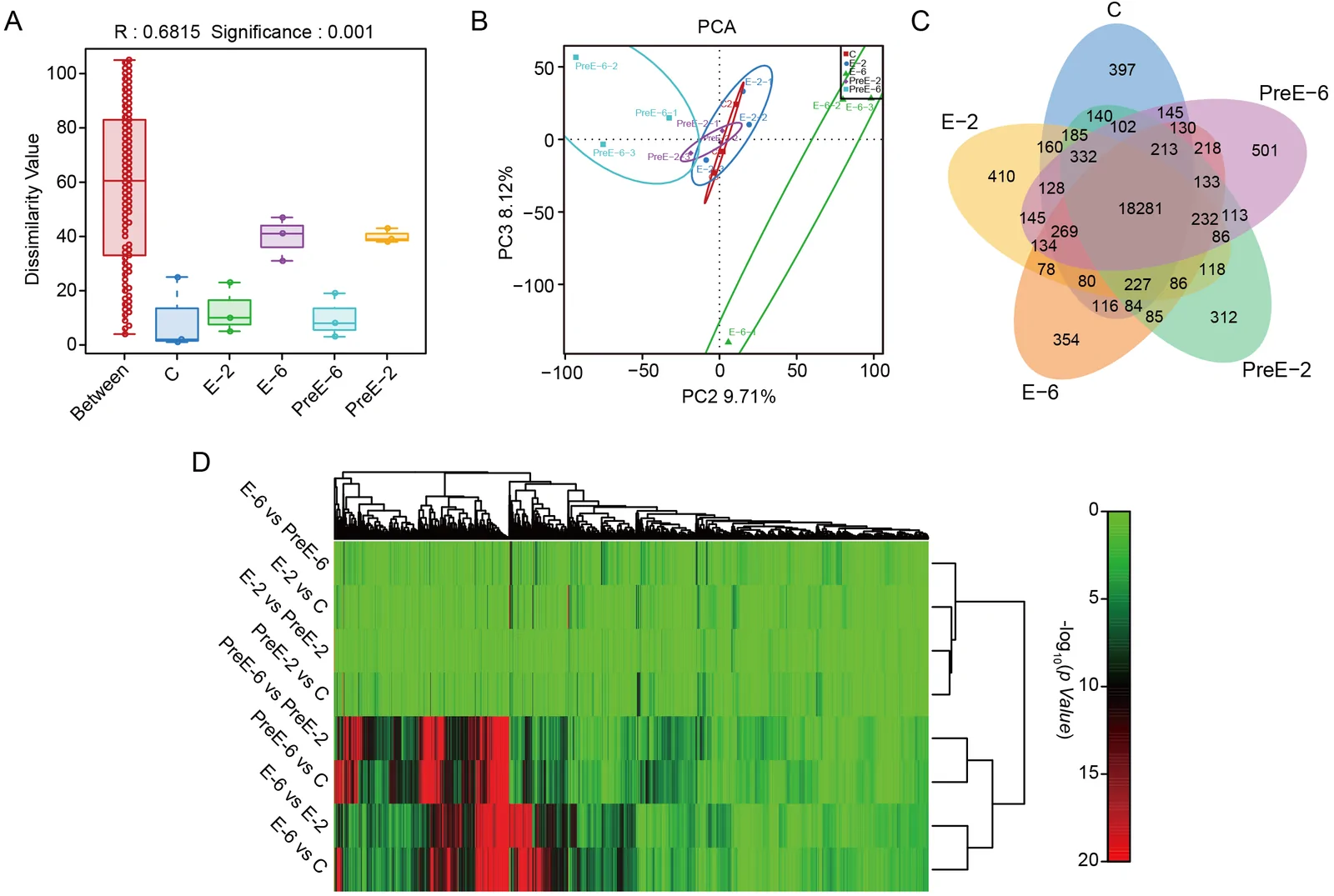
DEGs identified among different groups. **(A)** ANOSIM showing the discrepancies among groups. **(B)** PCA illustrating the overall differences across samples. **(C)** Venn diagram displaying the distribution of DEGs among groups. **(D)** Clustering heatmap of DEGs across different treatment groups.

At the transcriptional level, massive transcriptomic changes were triggered at 6 dpi (E6 and PreE6 groups), with thousands of DEGs identified when compared against control or 2-dpi infection groups. By contrast, far fewer DEGs were detected at the early infection stage (2 dpi), reflecting limited transcriptomic perturbation at this time point. Therefore, subsequent functional enrichment analyses were mainly focused on the 6 dpi samples. Detailed numbers of up- and down-regulated DEGs for each pairwise comparison are provided in **Supplementary Table S1**. The clustering heatmap of common DEGs exhibited treatment- and time-specific clustering patterns of core DEGs across comparison groups, suggesting that 6 dpi serves as a critical regulatory node for host cecal mucosal immune responses triggered by *E. tenella* infection (**Figure 2D**). Seven differentially expressed genes, including both upregulated and downregulated genes, were randomly selected from the transcriptomic dataset for quantitative real time PCR validation. As shown in **Supplementary Figure S1**, the expression trends of *PPARG*, *PLIN1*, *CYP1A1*, *THBS1*, *FMO4*, *CYP2C18*, and *CXCL14* were highly consistent with the RNA seq results, confirming the reliability and accuracy of the transcriptomic data obtained in this study.

### Transcriptomic analysis of host intestinal responses

GO enrichment analysis revealed that the DEGs identified from both precocious line comparisons (PreE6 vs C, PreE6 vs PreE2) and parent strain comparisons (E6 vs C, E6 vs E2) were predominantly enriched in cellular component terms including plasma membrane, membrane, and extracellular region. In terms of biological processes, DEGs from all comparison groups were commonly annotated to immune response, defense response, and cytokine production (**Figures 3A–D**). Specifically, DEGs in the PreE6 group were prominently enriched in leukocyte activation and T cell activation, whereas DEGs in the E6 group were additionally enriched in cytokine mediated signaling pathways, cytokine responses, and cellular responses to cytokine stimulation. KEGG pathway enrichment further verified that 6 dpi represents a critical response node dominated by multiple core immune signaling pathways. Both PreE6 vs C and E6 vs C comparisons exhibited significant enrichment in Th17 cell differentiation, NF - κB signaling pathway, intestinal immune network for IgA production, complement and coagulation cascades, Th1/Th2 cell differentiation, and natural killer cell mediated cytotoxicity (**Figures 3E, F**). Notably, the PreE6 group showed unique enrichment in the PPAR signaling pathway, chemokine signaling pathway, and leukocyte transendothelial migration pathway. In contrast, the E6 group was specifically enriched in steroid hormone biosynthesis, ECM receptor interaction, and fatty acid elongation pathways.

**Figure 3:**
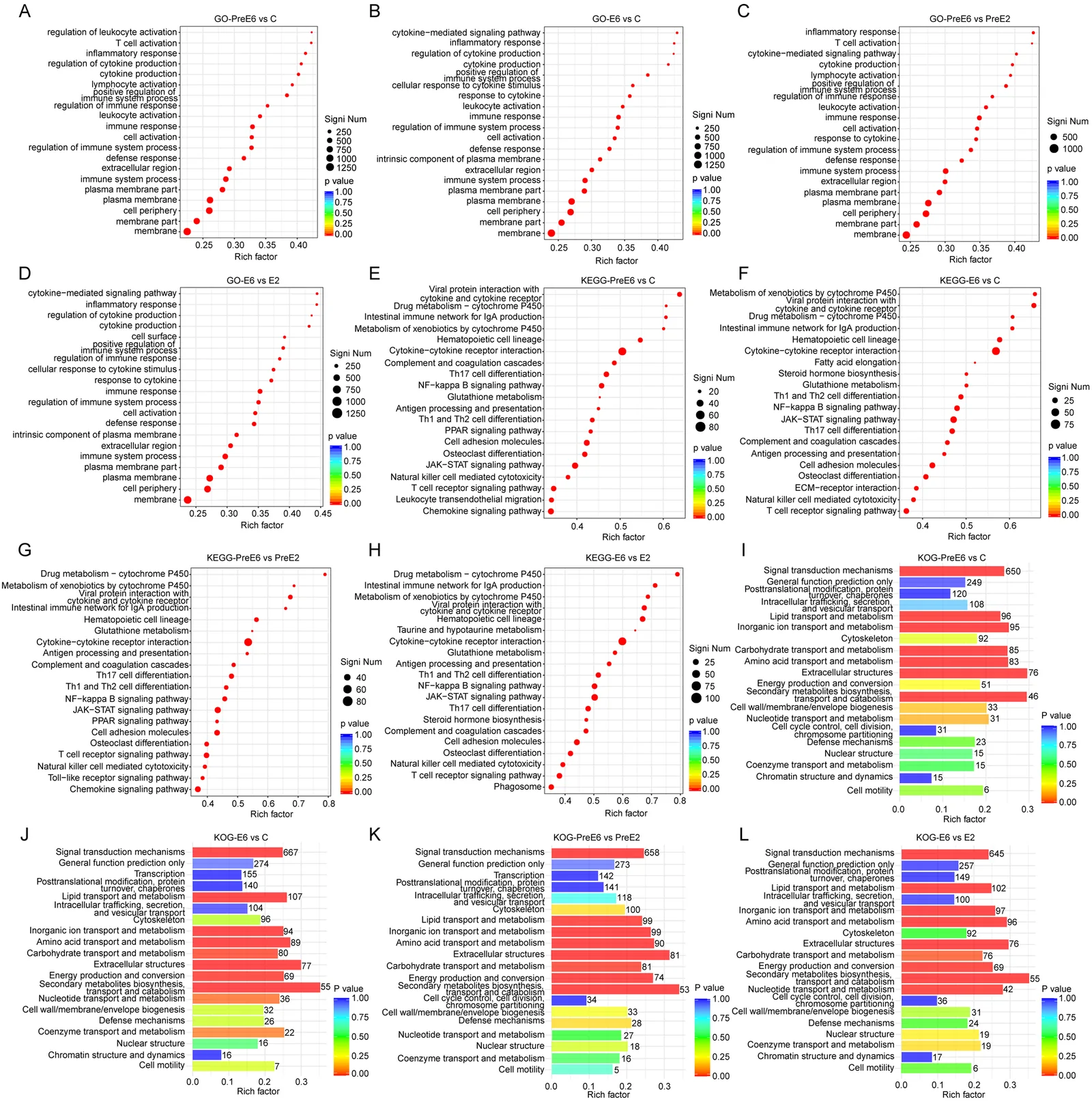
Transcriptome sequencing analysis of chick cecal tissues at different time points after infection *Eimeria* precocious line and parent strain. **(A–D)** Top 20 enriched GO terms of DEGs in the comparisons of PreE6 vs C, E6 vs C, PreE6 vs PreE2, and E6 vs E2. **(E–H)** Top 20 enriched KEGG pathways of DEGs in the comparisons of PreE6 vs C, E6 vs C, PreE6 vs PreE2, and E6 vs E2. **(I–L)** Top 20 enriched KOG classifications of DEGs in the comparisons of PreE6 vs C, E6 vs C, PreE6 vs PreE2, and E6 vs E2. PreE: precocious line oocyst infection; E: parent strain oocyst infection; C: blank control group.

Compared with the transcriptional profiles at 2 dpi, DEGs at 6 dpi in the PreE6 group were uniquely enriched in chemokine signaling, PPAR signaling, and Toll - like receptor signaling pathways, while DEGs in the E6 group were specifically enriched in phagosome, steroid hormone biosynthesis, and taurine and hypotaurine metabolism pathways (**Figures 3G, H**). KOG functional classification further supported these transcriptional trends. The DEGs were mainly categorized into extracellular structures, secondary metabolite biosynthesis and metabolism, lipid and amino acid transport and metabolism, and signal transduction mechanisms, indicating functional convergence centered on immune activation and metabolic remodeling (**Figures 3I–L**). To fully interpret transcriptional changes at the early infection stage (2 dpi), we displayed the top 20 significantly enriched GO terms, KEGG pathways and KOG classifications of DEGs from PreE2 vs C, E2 vs C, E2 vs PreE2 and E6 vs PreE6 comparisons (**Supplementary Figure S2**).

### Transcriptomic analysis of *Eimeria tenella*

To fully elucidate the linkage between strain developmental differences and host immune responses, differentially expressed genes of parasites within samples were synchronously annotated in this study. Functional enrichment analysis of parasite genes revealed that genes in the parasite from PreE6 group (P - PreE6) and parasite from E6 group (P - E6) were significantly enriched in catabolic processes and cellular component organization, respectively (**Figures 4A, B**). Volcano plot analysis identified 271 significantly upregulated genes and 116 significantly downregulated genes in the P - E6 vs P - PreE6 comparison group (**Figure 4C**). GO enrichment analysis showed that the parasite-derived DEGs in the P - E6 group, compared with the P - PreE6 group, were significantly downregulated in cellular component terms including membrane, plasma membrane, and extracellular region, and participated in biological processes such as cytokine response, external stimulus response, and type I interferon response (**Figure 4D**). KEGG pathway analysis indicated that the DEGs were upregulated in cytokine - cytokine receptor interaction, p53 signaling pathway, RIG - I - like receptor signaling pathway, and biosynthesis-related pathways, while they were downregulated in neuroactive ligand - receptor interaction, absorption - related pathways, and the Hedgehog signaling pathway (**Figure 4E**). KOG functional classification further verified that these DEGs were prominently enriched in signal transduction mechanisms and extracellular structure - related categories (**Figure 4F**).

**Figure 4:**
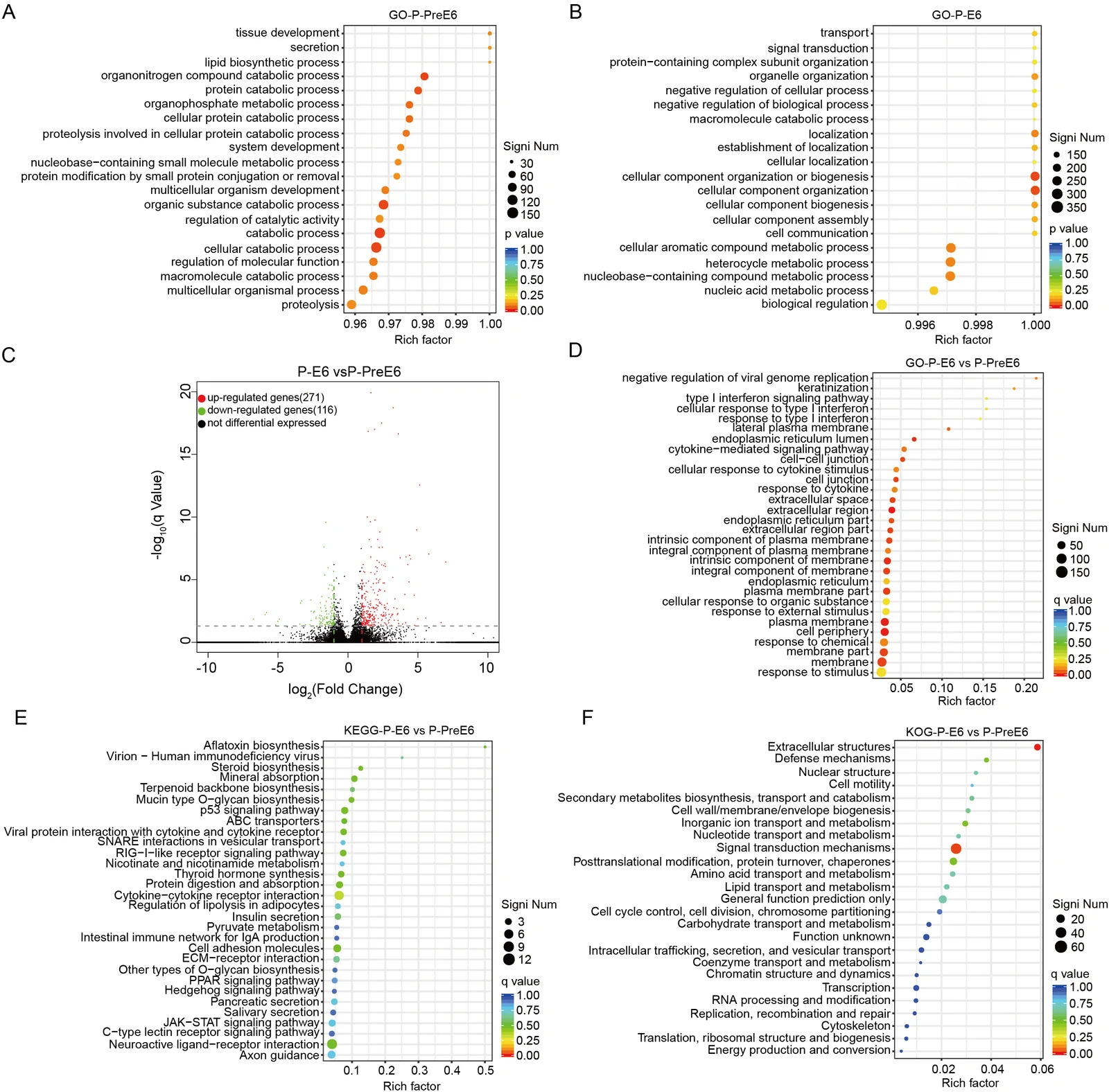
Transcriptomic analysis of *E. tenella* precocious line and parent strain. **(A)** Top 20 enriched GO terms of parasite genes in the *E. tenella* precocious line. **(B)** Top 20 enriched GO terms of parasite genes in the *E. tenella* parent strain. **(C)** Volcano plot showing DEGs between the P-E6 and P-PreE6 groups. **(D)** Top 30 enriched GO terms of DEGs between the P-E6 and P-PreE6 groups. **(E)** Top 30 enriched KEGG pathways of DEGs between the P-E6 and P-PreE6 groups. **(F)** Top 30 enriched KOG classifications of DEGs between the P-E6 and P-PreE6 groups. P-PreE: *E. tenella* precocious line oocysts; P-E: *E. tenella* parent strain oocysts.

### Clinical symptoms and pathological changes of cecal tissues

Gross anatomical observation and histological examination revealed rapid and severe cecal lesions in chicks infected with the parent strain. At 2 dpi, pinpoint hemorrhages and dark brown watery feces were observed in the ceca. Histologically, mild shortening of intestinal villi, shallow crypts, and obvious leukocyte infiltration in the lamina propria were detected. By 6 dpi, bloody cecal cores were formed in the intestinal lumen, accompanied by enlarged and blackened ceca with the intestinal wall thickened 2–3 times. Severe pan-necrosis and exfoliation of villous epithelium, massive oocyst accumulation in crypts and lamina propria, and extensive hemorrhage were observed in parent strain - infected tissues.

In contrast, the precocious line induced only mild pathological lesions. At 2 dpi, focal mild epithelial necrosis, mucosal hyperplasia, and intensive leukocyte infiltration in the lamina propria were observed. At 6 dpi, only moderate villus shortening and gametocyte - stage oocysts were detected, without diffuse hemorrhage or destruction of intestinal structural integrity (**Figure 5**). In summary, the parent strain exhibited rapid pathogenic progression and severe tissue damage, whereas the precocious line displayed remarkably attenuated virulence and controllable pathological lesions.

**Figure 5:**
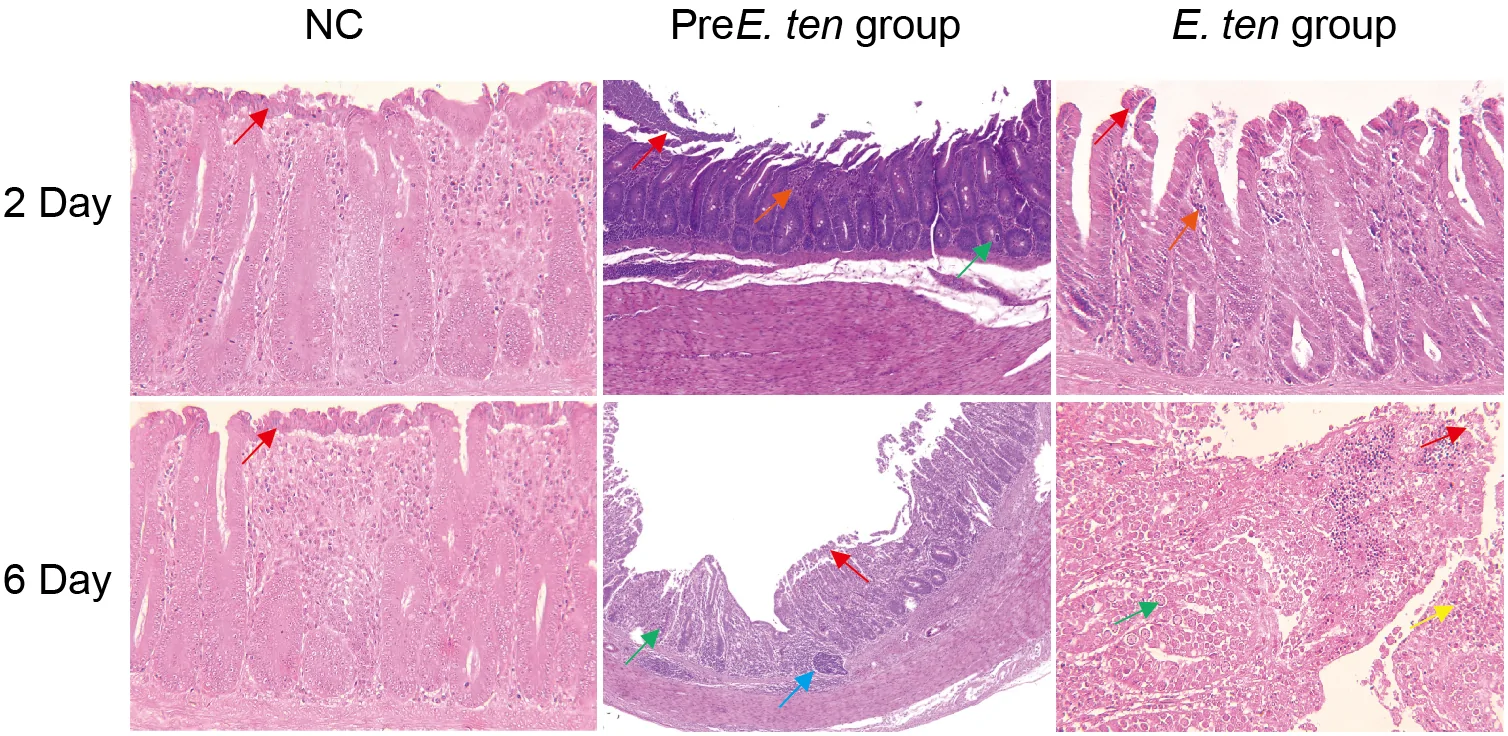
Histopathological sections of chick cecal tissues at 2 and 6 days after infection with different *E. tenella* strains. Red arrows: mucosal epithelial cells; orange arrows: inflammatory infiltration; green arrows: coccidial oocysts; blue arrows: lymphoid hyperplasia; yellow arrows: hemorrhage.

### Analysis of local mucosal immune levels in cecal tissues

MTS assay results showed that the proliferative activity of cecal lymphocytes in all infection groups was significantly higher than that in the control group and increased progressively with infection time, with significantly higher proliferation levels at 6 dpi than at 2 dpi. Notably, the lymphocyte proliferation ability in the parent strain-infected group was markedly higher than that in the precocious line group (**Supplementary Figure S3A**). Detection of cecal secretory immunoglobulin A (SIgA) indicated that SIgA levels in both parent and precocious line groups were significantly elevated at 2 dpi compared with the control group, suggesting that mucosal immunity was rapidly activated at the early infection stage. Moreover, SIgA contents were further increased at 6 dpi relative to 2 dpi, demonstrating a continuous enhancement of mucosal immune responses against coccidial infection (**Supplementary Figure S3B**).

### Immune protection assay

To further verify the immune protective effect of low-dose pre-immunization with *E. tenella* precocious line and parent strain, we performed an independent immune protection experiment via secondary virulent strain challenge. The clinical symptoms, growth performance, oocyst shedding, and immune organ development of chicks were comprehensively evaluated to systematically assess the protective efficacy of pre-immunization. No mortality was observed in all immunized groups on day 7 after secondary challenge with 1 × 10^5^ parent strain oocysts (**Figure 6A**). Although the body weight gain of all immunized groups remained significantly lower than that of the NC group, it was markedly higher than that of the PC group (**Figure 6B**). As shown in **Table 3**, bloody stool symptoms were still present in all immunized groups, but the bloody stool scores were significantly lower than those in the PC group. Compared with the PC group, chicks pre-immunized with 1 × 10^3^ and 1 × 10^4^ *E. tenella* oocysts (Pre*E. ten* and *E. ten* groups) exhibited significantly reduced cecal OPG values after secondary challenge (**Figure 6C**). Immune organ index analysis showed that the thymus indices of the 1 × 10^3^ immunized group and the 1 × 10^4^ precocious line immunized group were higher than that of the PC group. The bursa of Fabricius index was also significantly increased in the 1 × 10^4^ parent strain immunized group, while no significant difference in spleen index was detected among all groups (**Figure 6D**). In conclusion, pre - immunization with low - dose precocious line and parent strain oocysts can induce effective immune protection, significantly alleviate intestinal lesions, reduce oocyst shedding and mortality after secondary infection, and exert positive effects on the development of immune organs in chicks.

**Figure 6:**
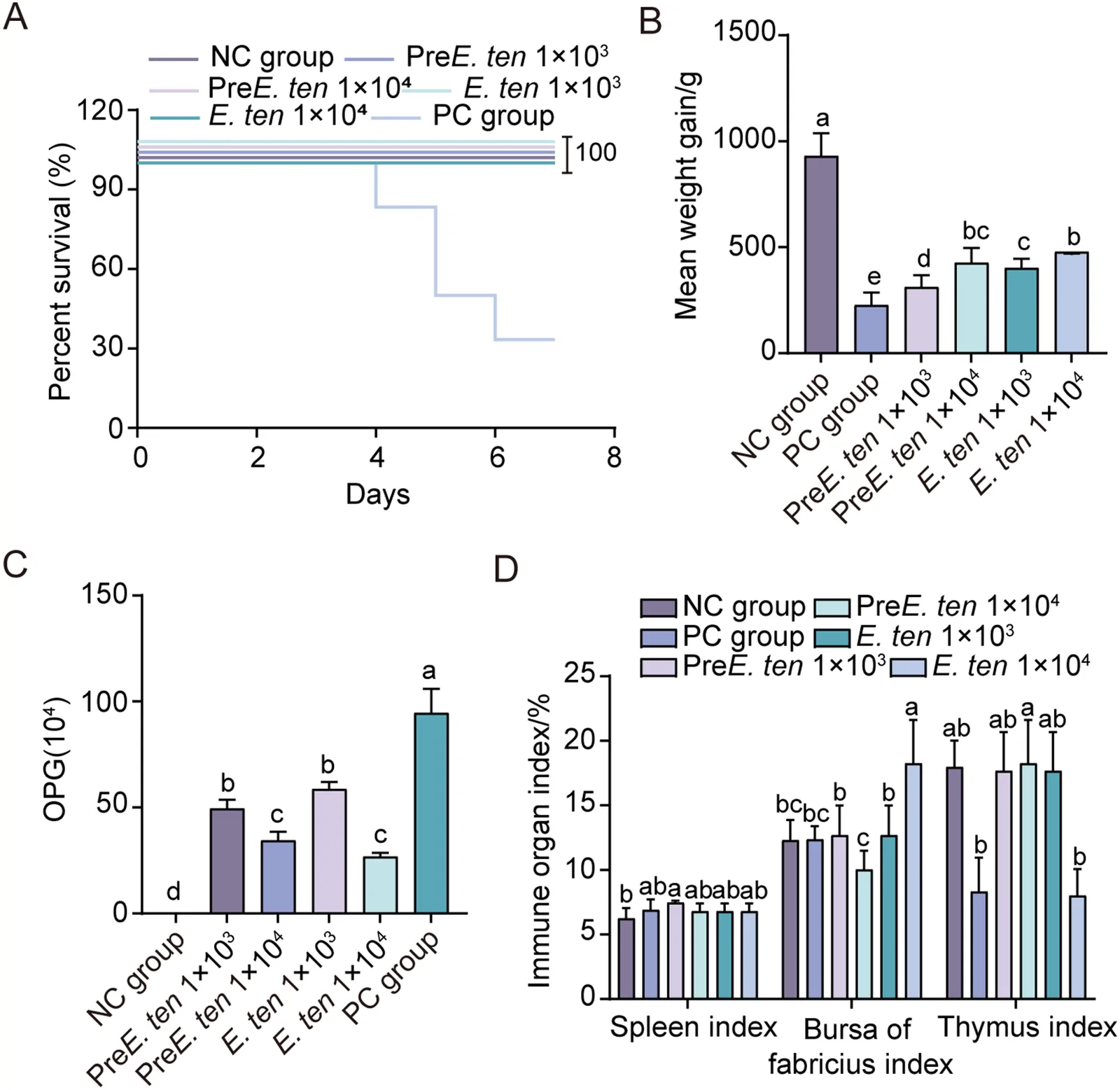
Immune protection assay of sporulated oocysts of *E. tenella* precocious line and parent strain. **(A)** Effects of different dosages of precocious line and parent strain oocysts on chick survival rate during immune protection detection. **(B)** Effects of different dosages of precocious line and parent strain oocysts on chick body weight gain during immune protection detection. **(C)** OPG of cecal contents in each group in the immune protection assay. **(D)** Changes of immune organ indices in each group. Values marked with different lowercase superscript letters (a-d) within the same column indicate significant differences at *P* < 0.05.

**Table 3:** Statistical analysis of bloody stool scores after secondary infection.

Group	Oocyst infection dose	84 h	93 h	108 h	117 h	132 h	141 h	156 h	165 h	Score of bloody feces
NC	0	0	0	0	0	0	0	0	0	0^c^
PC	–	4	4	4	3	3	2	0	0	2.5 ± 1.69^a^
Pre*E. ten*	1.0×10^3^	2	3	2	1	1	0	0	0	1.13 ± 1.13^b^
Pre*E. ten*	1.0×10^4^	0	2	1	0	1	0	0	0	0.5 ± 0.76^bc^
*E. ten*	1.0×10^3^	1	3	1	1	0	0	0	0	0.75 ± 1.04^bc^
*E. ten*	1.0×10^4^	0	1	0	0	0	0	0	0	0.13 ± 0.35^bc^

Data are presented as mean ± SE (n = 6); Values marked with different lowercase superscript letters (a-d) within the same column indicate significant differences at *P* < 0.05.

## Discussion

Pathogenicity assays confirmed obvious dose-dependent virulence differences between the parental strain and the precocious line. High-dose infection with the parental strain caused substantial mortality in chicks, whereas the precocious line exhibited markedly attenuated virulence across all challenge doses. These *in-vivo* pathological phenotypes provide phenotypic support for our subsequent molecular-level analyses in this study. Importantly, results from the pathogenicity test guided our selection of 1 × 10^4^ sporulated oocysts as the standardized challenge dose for transcriptome sequencing; this dose could establish stable infection while avoiding massive mortality that would interfere with tissue sampling.

In the present study, temporal transcriptomic profiling of chick cecal tissues was performed at two critical developmental stages of *E. tenella*, schizogony (2 dpi) and gametogony (6 dpi), to systematically compare the differential host molecular responses triggered by virulent parental strain and attenuated precocious line infections. Transcriptomic analysis revealed that only approximately 200 differentially expressed genes (DEGs) were identified at 2 dpi, reflecting mild global transcriptional remodeling of cecal tissues during the early infection phase. In contrast, the number of DEGs increased sharply to 2000–3500 at the gametogony stage (6 dpi), indicating that gametogony acts as the core stage for activating cecal mucosal immune responses in chicks. Accordingly, subsequent functional enrichment analyses in this study primarily focused on the transcriptomic data obtained at 6 dpi. Although both strains activated the upstream cytokine-JAK/STAT innate immune pathway, their downstream signaling cascades exhibited distinct divergence, driving two different regulatory patterns: destructive inflammation and balanced immune homeostasis.

The parental strain triggered activation of the NF-κB signaling pathway, which simultaneously activated three detrimental biological cascades, including coagulation-complement-extracellular matrix (ECM) positive feedback loops, steroid metabolic disorders, and antioxidant system depletion, which likely contribute to severe cecal tissue lesions. Previous studies have demonstrated that inhibition of the NF-κB pathway effectively relieves *Eimeria*-induced intestinal damage, confirming the crucial pathogenic function of NF-κB signaling during avian coccidiosis (13). Sustained NF-κB hyperactivation promotes the robust secretion of pro-inflammatory mediators and upregulates tissue factors to initiate the extrinsic coagulation cascade. Complement activation produces C3a and C5a, which recruit inflammatory infiltrating cells, while activated coagulation products further amplify complement responses, forming a positive inflammation–thrombosis feedback loop. This cascade continuously degrades extracellular matrix components and impairs intestinal epithelial barrier integrity, which is consistent with previous reports on EtROP17-mediated cell injury and inflammatory bowel disease-associated mechanisms (14–16).Meanwhile, severe infection may trigger host stress disturbance accompanied by aberrant activation of the hypothalamic-pituitary-adrenal axis, where putative excessive steroid secretion could suppress Th1/Th17-mediated protective immunity and impair host parasite-clearing capacity, and sustained oxidative stress might lead to massive taurine consumption, antioxidant system exhaustion and reactive oxygen species accumulation, which in turn could potentially accelerate intestinal epithelial cell necrosis (17–19). The microscopic pathological features of pinpoint cecal hemorrhage at 2 dpi and extensive cecal core formation with mucosal exfoliation at 6 dpi are the intuitive morphological manifestations of this progressive pathological cascade.

In marked contrast to the destructive regulatory pattern induced by the parental strain, the precocious line specifically activates PPAR signaling, TLR signaling, and leukocyte transendothelial migration pathways, establishing an integrated regulatory cascade of “immune sensing-inflammatory restriction-pathogen elimination-tissue repair” to balance anti-parasite immune defense and intestinal immune homeostasis. PPAR-γ was identified as the most distinctive core molecular marker distinguishing precocious line infection from parental strain infection, with significant pathway enrichment detected as early as 2 dpi. Notably, PPAR-γ pathway activation represents a targeted early regulatory response rather than widespread transcriptomic perturbation, which reasonably explains the mild global transcriptional changes observed at 2 dpi. Mechanistically, PPAR-γ binds to the NF-κB p65 subunit and upregulates IκBα expression, thereby blocking NF-κB nuclear translocation and suppressing the excessive secretion of pro-inflammatory cytokines such as TNF-α and IL-6, which mediates early lipid-dependent anti-inflammatory effects (20). TLR signaling exhibits precise temporal activation during precocious line infection: chTLR7/15 is activated in the early stage to initiate innate immune responses, while chTLR21 sustains continuous immune signaling throughout the infection period. This temporal activation mode induces chemokine-mediated directional leukocyte migration to infected sites for parasite elimination, accompanied by the secretion of anti-inflammatory cytokines (IL-10, TGF-β) to promote mucosal repair and prevent TLR overactivation-induced cytokine storms (21). This refined regulatory mechanism only triggers mild local mucosal injury, without diffuse hemorrhage or full-thickness epithelial necrosis.

Based on the systematic comparison of differential pathway activation between the two strains, this study constructed an integrated mechanistic model to elucidate the divergent pathogenic mechanisms of parental and precocious *E. tenella* strains (**Figure 7**). Both strains share the upstream NF-κB-dependent innate immune initiation signal. However, the precocious line harbors an early PPAR-γ-mediated anti-inflammatory module that negatively restrains excessive NF-κB activation and inflammatory overreaction. In comparison, the parental strain lacks this negative regulatory mechanism, and persistent NF-κB hyperactivation drives multiple pathological cascades, ultimately resulting in the destruction of intestinal structural integrity. These divergent regulatory patterns form a unique bipolar immune regulatory paradigm during *E. tenella* infection.

**Figure 7:**
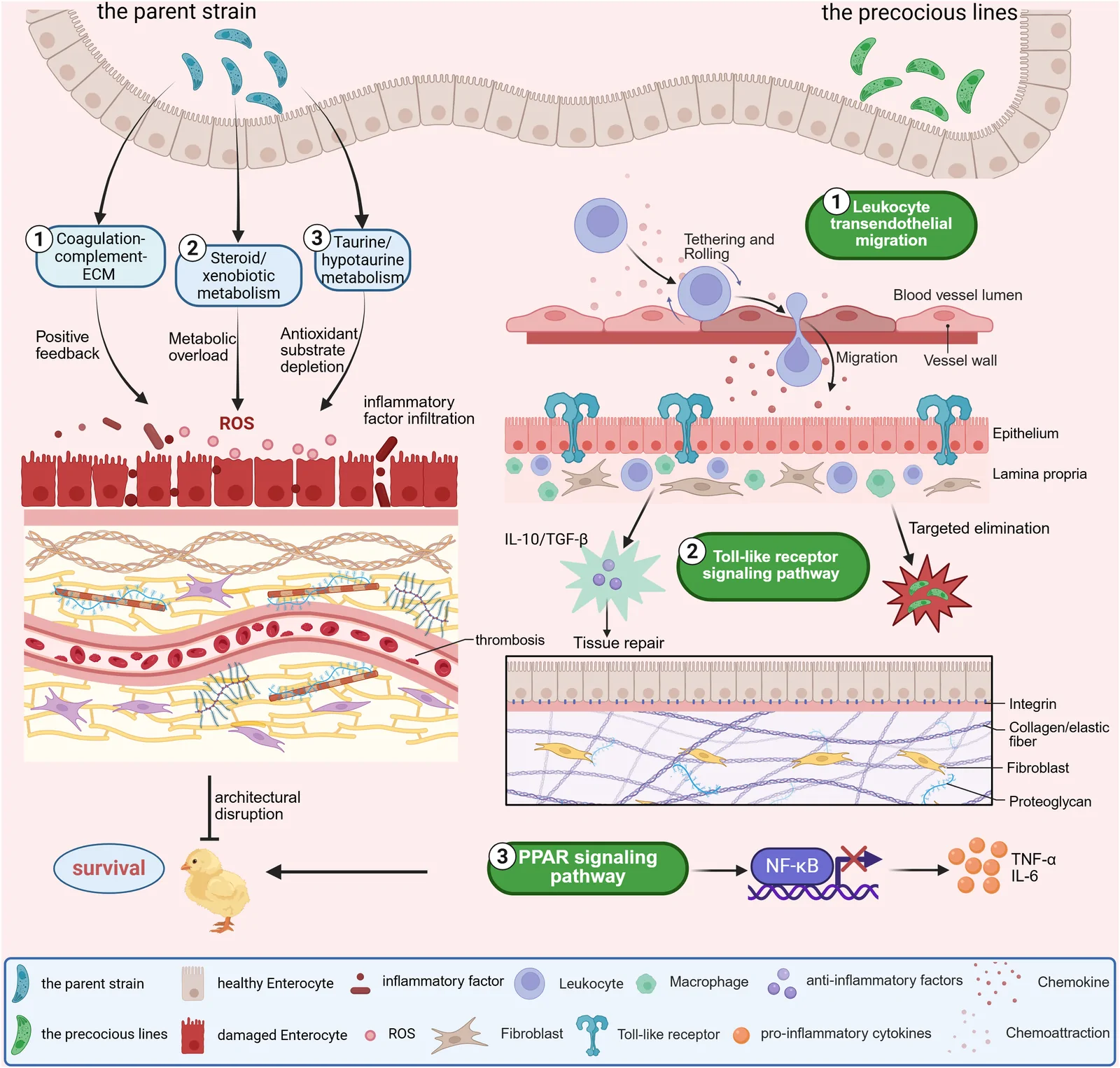
Schematic diagram of the differential pathogenic mechanisms in chicks infected with *E. tenella* precocious line and parent strain oocysts. The parental strain simultaneously activates three harmful pathways: (1) coagulation-complement-ECM pathway inducing thrombosis; (2) steroid hormone biosynthesis pathway causing host metabolic disturbance; (3) taurine/hypotaurine metabolism pathway leading to antioxidant system depletion. Combined damages likely result in full intestinal-architecture disruption. In contrast, the precocious line triggers early activation of three protective pathways: (1) leukocyte transendothelial migration pathway recruiting immune cells for targeted pathogen clearance; (2) Toll-like receptor signaling pathway initiating local immunity and facilitating tissue repair via IL-10 and TGF-β; (3) PPAR signaling pathway that negatively regulates NF-κB activation to limit pro-inflammatory TNF-α and IL-6 production and avert excessive tissue damage. These two regulatory modes exhibit sharply contrasting outcomes. The figure is created with BioRender.com.

Transcriptomic data of *E. tenella* parasites revealed that oocysts of the parental strain may maintain high pathogenicity by elevating metabolic activity and the expression of invasion-associated proteins. This finding explains the molecular mechanism underlying the low fecundity of the precocious line from the pathogen perspective and provides theoretical support for its application as a vaccine-candidate strain. Measurements of cecal lymphocyte proliferation capacity and mucosal SIgA levels confirmed that both the precocious line and parental strain of *E. tenella* can effectively activate intestinal mucosal immune cells and strengthen mucosal secretory immune function, which supplies critical immunological evidence for the subsequent immune-protective outcomes. In the immune-protection assay, chicks in all immunized groups exhibited alleviated bloody-stool symptoms, reduced oocyst shedding, improved development of immune organs and zero mortality after secondary challenge, demonstrating that both strains are capable of inducing effective anti-coccidial protective immunity despite their distinct virulence profiles.

Although precocious attenuated strains have been widely applied for the commercial prevention and control of avian coccidiosis, most existing studies focus predominantly on parasite-derived virulence genes, while systematic temporal profiling of host mucosal immune responses across different developmental stages (schizogony and gametogony) remains limited. Based on dynamic transcriptomic data at 2 and 6 dpi, this study preliminarily identified PPAR-γ as the core hub for maintaining intestinal inflammatory homeostasis and clarified the strain-specific divergent inflammatory mechanisms, filling the research gap in the host-parasite temporal interaction mechanism during *E. tenella* infection.

Nevertheless, this study has several inherent limitations. First, all experiments were performed using White Leghorn layers, and whether the NF-κB/PPAR-γ regulatory mechanism identified in this study is applicable to broiler breeds requires further verification. Second, the crosstalk between NF-κB and PPAR-γ was analyzed based on *in vivo* transcriptomic association data, without validation at the cellular level. Third, pharmacological functional verification using PPAR-γ agonists or inhibitors was not conducted; thus, the causal relationship between PPAR-γ signaling and intestinal immune homeostasis during coccidial infection needs further functional confirmation. Finally, the 22-h prepatent-period difference between the parental and precocious lines arises from accelerated asexual schizogony in the precocious line. At 144 h post-infection, however, both strains still exhibited mixed populations of late-stage schizonts and gametocytes, differing only in stage-specific parasite proportions; the precocious line had not fully developed into oocysts. DEGs detected at 6 dpi in this study are attributable to both intrinsic strain genetic backgrounds and varying proportions of developmental stages, which cannot be fully distinguished using single-time-point samples. We therefore interpreted mainly DEGs with large expression changes and stable pathway functions, rather than subtle, highly stage-dependent transcriptomic alterations. Multi-time-point sampling and stage-resolved parasite transcriptomics in future work will help disentangle strain-specific molecular signatures from developmental-stage effects.

## Conclusion

Based on comparative temporal transcriptomic analysis of chick cecal tissues at 2 and 6 dpi, the present study preliminarily revealed that the pathogenic discrepancy between *E. tenella* parental strain and precocious line is governed by the bipolar divergence of host inflammatory regulatory pathways. The virulent parental strain induces persistent NF-κB hyperactivation, triggering a series of pathological cascades that lead to irreversible cecal tissue damage. In contrast, the precocious line significantly activates PPAR-γ signaling at the early infection stage, which cooperates with the TLR pathway to precisely balance anti-parasite immune defense and intestinal immune homeostasis. PPAR-γ was identified as the core molecular hub responsible for mild tissue damage and well-orchestrated mucosal immune responses during precocious line infection. As a commercially available live attenuated vaccine strain for avian coccidiosis prevention, the precocious line has been widely applied in poultry production. This study systematically elucidates the unique mucosal immune regulatory mechanism triggered by the precocious line, improves the theoretical framework for interpreting the pathogenic characteristics of attenuated *E. tenella* strains, and provides valuable molecular insights for the targeted and antibiotic-free prevention and control of avian coccidiosis.

## Data Availability

All raw sequencing reads generated in this study have been deposited in the NCBI Sequence Read Archive (SRA) under accession number PRJNA1491229. (https://www.ncbi.nlm.nih.gov/).

## References

[1] Nguyen BT Flores RA Kim T Min W . Understanding *Eimeria* infection for the treatment and prevention of chicken coccidian parasites. Front Cell Infect Microbiol. (2026) 16:1756128. doi: 10.3389/fcimb.2026.1756128 41969659 PMC13066156

[2] Liu G Choppa VSR Sharma MK Ko H Choi J Kim WK . Effects of methionine supplementation in a reduced protein diet on growth performance, oxidative status, intestinal health, oocyst shedding, and methionine and folate metabolism in broilers under *Eimeria* challenge. J Anim Sci Biotechnol. (2024) 15:84–109. doi: 10.1186/s40104-024-01041-4 38853257 PMC11163814

[3] Bafundo K . Current thinking on *Eimeria* spp. in poultry and potential for advancements in control. Anim (Basel). (2025) 15:424–9. doi: 10.3390/ani15030424 39943194 PMC11815899

[4] Ewais O Abdel-Tawab H El-Fayoumi H Aboelhadid SM Al-Quraishy S Falkowski P . Antioxidant properties of D-limonene and its nanoemulsion form enhance its anticoccidial efficiency in experimentally infected broilers with *Eimeria tenella*: an *in vitro* and *in vivo* study. Vet Res Commun. (2024) 48:3711–25. doi: 10.1007/s11259-024-10512-y 39235470

[5] Huang J Cheng K Wang J . An integrated model of biphasic apoptosis in avian coccidiosis: molecular networks and host-parasite interplay. Anim (Basel). (2025) 15:3528. doi: 10.3390/ani15243528 41463813 PMC12729945

[6] Li Y Suo J Liang R Liang L Liu X Ding J . Genetic manipulation for the non-model protozoan *Eimeria*: advancements, challenges, and future perspective. iScience. (2025) 28:112060. doi: 10.1016/j.isci.2025.112060 40109377 PMC11919594

[7] Yue H Lei XW Yang FL Li MY Tang C . Reference gene selection for normalization of PCR analysis in chicken embryo fibroblast infected with H5N1 AIV. Virol Sin. (2010) 25:425–31. doi: 10.1007/s12250-010-3114-4 21221921 PMC7090763

[8] Tu JH Liu BG Lin BJ Liu HC Guo SC Ouyang QY . Single-cell transcriptomic atlas of the chicken cecum reveals cellular responses and state shifts during *Eimeria tenella* infection. BMC Genomics. (2025) 26:141–51. doi: 10.1186/s12864-025-11302-9 39948469 PMC11827208

[9] Fan Q Wang Z Wang J Wang Y Zhang Z Yu L . Transcriptome-wide mapping of internal mRNA N^7^-methylguanosine in sporulated and unsporulated oocysts of *Eimeria tenella* reveals stage-specific signatures. Parasit Vectors. (2024) 17:491–500. doi: 10.1186/s13071-024-06580-3 39605027 PMC11603632

[10] Zhang L . Differences in Pathogenicity Between Precocious and Virulent Strains of Eimeria Tenella and Research on its Virulence Genes. Taigu, Shanxi, China: PhD Diss. Shanxi Agricultural University (2020).

[11] Youn HJ Noh JW . Screening of the anticoccidial effects of herb extracts against *Eimeria tenella*. Vet Parasitol. (2001) 96:257–63. doi: 10.1016/s0304-4017(01)00385-5 11267752

[12] Saber A Mawas AS Khalil AM Ahmed AI Shibat Elhamd DMW Ali E . Therapeutic impact of dietary ginger supplementation in chickens experimentally infected with coccidia-anti-oxidant, biochemical, and pathological evaluations. Front Vet Sci. (2025) 12:1511759. doi: 10.3389/fvets.2025.1511759 40230796 PMC11995867

[13] Meng X Gan X Wang Y Zhang Q Duan X Wang Y . Saikosaponin inhibits *Eimeria tenella* infection by modifying the NF-κB pathway and regulating cytokines and the intestinal microbial community. Exp Parasitol. (2024) 267:108861. doi: 10.1016/j.exppara.2024.108861 39536811

[14] Scaldaferri F Lancellotti S Pizzoferrato M De Cristofaro R . Haemostatic system in inflammatory bowel diseases: new players in gut inflammation. World J Gastroenterol. (2011) 17:594–608. doi: 10.3748/wjg.v17.i5.594 21350708 PMC3040331

[15] Meng YJ Mu BJ Liu XX Yu LM Zheng WB Xie SC . Transcriptional changes in LMH cells induced by *Eimeria tenella* rhoptry kinase family protein 17. Front Vet Sci. (2022) 9:956040. doi: 10.3389/fvets.2022.956040 36016802 PMC9395702

[16] Pryzdial ELG Leatherdale A Conway EM . Coagulation and complement: key innate defense participants in a seamless web. Front Immunol. (2022) 13:918775. doi: 10.3389/fcimb.2026.1756128 36016942 PMC9398469

[17] Raber J . Detrimental effects of chronic hypothalamic-pituitary-adrenal axis activation. From obesity to memory deficits. Mol Neurobiol. (1998) 18:1–22. doi: 10.1007/BF02741457 9824846

[18] Xu L Wang QZ Du XG Cao WP Tang ZR Zhang XL . Anti-inflammatory and antioxidant mechanisms of taurine and its application in young animals. Chin J Anim Nutr. (2015) 27:3683–8. Available online at: http://www.chinadoi.cn/searchDoi?querywords=%E7%89%9B%E7%A3%BA%E9%85%B8%E6%B6%88%E7%82%8E%E6%8A%97%E6%B0%A7%E5%8C%96%E6%9C%BA%E7%90%86%E5%8F%8A%E5%85%B6%E5%9C%A8%E5%B9%BC%E9%BE%84%E5%8A%A8%E7%89%A9%E4%B8%AD%E7%9A%84%E5%BA%94%E7%94%A8.

[19] Seneff S Kyriakopoulos AM . Taurine prevents mitochondrial dysfunction and protects mitochondria from reactive oxygen species and deuterium toxicity. Amino Acids. (2025) 57:6–20. doi: 10.1007/s00726-024-03440-3 39789296 PMC11717795

[20] Ju Z Su M Hong J Kim E Jung J . Anti-inflammatory effects of an optimized PPAR-γ agonist via NF-κB pathway inhibition. Bioorg Chem. (2020) 96:103611. doi: 10.1016/j.bioorg.2020.103611 32007723

[21] Wang S Wang D Bai Y Zheng G Han Y Wang L . Expression of Toll-like receptors and host defence peptides in the cecum of chicken challenged with *Eimeria tenella*. Parasite Immunol. (2024) 46:e13022. doi: 10.1111/pim.13022 38384176

