# Supplementary material for: Transcriptomic analysis of cecal mucosal immunity in SPF White Leghorn chicks infected with precocious and parent strains of Eimeria tenella
Source: Front Immunol. 2026 Sep 28;17:1954459. doi: 10.3389/fimmu.2026.1954459 (PMC13655785; doi:10.3389/fimmu.2026.1954459)
Supplement: Supplementary file 1 [file Table1.docx]

**Table S1**

Numbers of significantly up- and down-regulated DEGs in each pairwise comparison group.

| Comparison group | Up-regulated DEGs | Down-regulated DEGs |
| --- | --- | --- |
| E6 vs C | 2004 | 1555 |
| PreE6 vs C | 1781 | 1557 |
| E6 vs E2 | 2053 | 1432 |
| PreE6 vs PreE2 | 1903 | 1547 |
| E2 vs PreE2 | 16 | 9 |
| PreE2 vs C | 109 | 113 |
| E2 vs C | 79 | 88 |
| E6 vs PreE6 | 271 | 116 |
